# Sampling of healthcare professionals’ perspective on point-of-care technologies from 2019–2021: A survey of benefits, concerns, and development

**DOI:** 10.1371/journal.pone.0299516

**Published:** 2024-03-08

**Authors:** Taylor Orwig, Shiv Sutaria, Ziyue Wang, Sakeina Howard-Wilson, Denise Dunlap, Craig M. Lilly, Bryan Buchholz, David D. McManus, Nathaniel Hafer

**Affiliations:** 1 Department of Medicine, UMass Chan Medical School, Worcester, MA, United States of America; 2 Manning School of Business, UMass Lowell, Lowell, MA, United States of America; 3 Department of Anesthesiology and Perioperative Medicine, UMass Chan Medical School, Worcester, Massachusetts, United States of America; 4 Department of Surgery, UMass Chan Medical School, Worcester, Massachusetts, United States of America; 5 Department of Biomedical Engineering, UMass Lowell, Lowell, MA, United States of America; 6 Department of Population and Quantitative Health Sciences, UMass Chan Medical School, Worcester, MA, United States of America; 7 UMass Center for Clinical and Translational Science, UMass Chan Medical School, Worcester, MA, United States of America; 8 Program in Molecular Medicine, UMass Chan Medical School, Worcester, MA, United States of America; Zambia Ministry of Health, ZAMBIA

## Abstract

Point-of-care technology (POCT) plays a vital role in modern healthcare by providing a fast diagnosis, improving patient management, and extending healthcare access to remote and resource-limited areas. The objective of this study was to understand how healthcare professionals in the United States perceived POCTs during 2019–2021 to assess the decision-making process of implementing these newer technologies into everyday practice. A 5-point Likert scale survey was sent to respondents to evaluate their perceptions of benefits, concerns, characteristics, and development of point-of-care technologies. The 2021 survey was distributed November 1^st^, 2021- February 15th, 2022, with a total of 168 independent survey responses received. Of the respondents, 59% identified as male, 73% were white, and 48% have been in practice for over 20 years. The results showed that most agreed that POCTs improve patient management (94%) and improve clinician confidence in decision making (92%). Healthcare professionals were most concerned with potentially not being reimbursed for the cost of the POCT (37%). When asked to rank the top 3 important characteristics of POCT, respondents chose accuracy, ease of use, and availability. It is important to note this survey was conducted during the COVID-19 pandemic. To achieve an even greater representation of healthcare professionals’ point of view on POCTs, further work to obtain responses from a larger, more diverse population of providers is needed.

## Introduction

Realizing the full potential of point-of-care technologies (POCTs) represents a critical factor in advancing health care by making the predictive, preemptive, preventive, and personalized care more accessible [1]. Point-of-care tests are defined as tests that can be performed onsite or at the bedside in clinics, Emergency Rooms (ERs), and at home and must provide results without delay. Some examples of POCT include blood glucose tests for diabetes, home pregnancy tests, automatic blood pressure cuffs, or antigen-based-over-the-counter diagnostics for SARS-CoV-2.

The COVID-19 pandemic demonstrated the urgent need for accessible, rapid, and accurate testing [2–6]. This also led to a challenge in access to healthcare, specifically, scheduling annual check-ups and wellness visits with providers. As a result, health concerns were pushed off or managed using telehealth [7]. In this context, POCT provides new ways to support diagnostics and help patients monitor their treatments.

To gain a comprehensive understanding of the utilization of POCTs in current medical practice and identify opportunities for improvement, surveys are a valuable tool to assess the perspectives of clinicians and patients and identify POCT advantages and drawbacks [8,9]. Point-of-care tests are frequently utilized by healthcare professionals to obtain real-time, accurate patient data, making it crucial to comprehend their perspectives on POCT. The aim in reintroducing the clinician focused POCT survey for the third consecutive year was to continue identifying areas of need and foster new research and development, thereby directly impacting future production and implementation of POCT devices. Recent years have witnessed significant advancements in point-of-care technology [10], empowering healthcare professionals to conduct rapid diagnostic tests and make prompt treatment decisions at the patient’s bedside or in other clinical settings. As these technologies evolve, understanding how they are used enhances the development process. To analyze whether opinions regarding POCTs have changed or remained consistent over time, the results of the 2021 survey were compared with previous years findings.

## Materials and methods

The 2021 POCT survey was developed using the same methodologies used in years 2019 and 2020 [8,9]. This survey was distributed to a diverse group of healthcare professionals to assess their views on the importance and quality of point-of-care devices. The group consisted of clinicians, researchers, and product developers. Like previous years, the 2021 survey was sent primarily by email but was also advertised on LinkedIn. Similar contacts from past surveys were obtained from large directories including University of Massachusetts Center for Clinical Translational Science (UMCCTS), Massachusetts Medical Device Development Center (M2D2), UMass Memorial Health (UMMH), Consortia for Improving Medicine with Innovation & Technology (CIMIT), Center for Advancing Point of Care Technologies (CAPCaT), National Center for Complementary and Integrative Health (NCCIH), and other directories within the National Heart, Lung, and Blood Institute including, Small business Research Initiative (SBIR), NIH Center for Accelerated Innovations (NCAI), and Research Evaluation and Commercialization Health (REACH) mailing lists. The exact number of individuals who opened the emails is unknown but is thought to be greater than 15,000. If potential respondents did not initially reply, a reminder email was sent in the following weeks. The survey was launched 01 November 2021 and closed 15 February 2022. The only exclusion criteria were not self-identifying as a healthcare worker. A total of 168 responses were received. This study was deemed to be exempt from review by the Institutional Review Board (IRB) in July 2019 by the UMass Chan Medical School’s IRB (docket#H00018195).

### Survey development, data collection, and storage

In 2019, CAPCaT investigators convened a panel including survey researchers, clinicians, and business development experts to develop a survey focused on assessing provider impressions of POCT [8,9]. In 2020, questions were added to assess the impact of COVID-19 on healthcare provider impressions of POCT. In most cases, the questions listed in this survey were identical to questions asked in the 2019 survey. In 2021, the COVID-19 section was removed and several new questions were added to address specific issues that have emerged since the start of the COVID-19 pandemic. Due to certain questions being omitted or introduced over the years, statistical analyses were used only on questions that were identical in the 2019–2021 surveys (see S1 File).

The clinician POCT survey included items regarding the benefits, concerns, characteristics, and development of POCTs including business and strategic styles of practice. The questions measuring general POCT matters were adapted from the National Heart, Lung, and Blood Institute (NHLBI) strategic vision published in 2016 [11], and from a survey developed by researchers from the Point-of-Care Technology Research Network (POCTRN) center located at Johns Hopkins University [12]. Questions regarding business-related aspects of healthcare technology were adapted from two seminal studies focused on the adoption of new technologies [13,14]. The full survey instrument can be found in the S1 File.

Most survey items used a Likert-like scale that allowed participants to select “strongly disagree,” “disagree,” “neutral,” “agree,” or “strongly agree.” Demographic information was collected via multiple-choice questions or through open-ended text boxes. Participants were further asked to list up to five conditions for which POCT could help with: (1) diagnosis of a disease, and (2) management or monitoring of a disease. Participants’ answers to these two questions were through open-ended textboxes.

The survey was generated using a REDCap (Research Electronic Data Capture) interface. All data were received from participants and transmitted directly into the study server for storage [15,16]. The secure server is hosted by the UMass Chan network and was only accessed by authorized individuals.

#### Data analysis

The variables from questions that employed the 5-point Likert-like scale described above were collected into two categories: (1) responses indicating “strongly agree” and “agree” were categorized into agreement, and (2) “strongly disagree” and “disagree” were designated as disagreement. Any “neutral” response was excluded from the analysis. Analysis of important characteristics of POCTs was determined using the following point system; 1st most important = 3 points, 2nd most important = 2 points, and 3rd most important = 1 point. Calculating data from survey respondents was limited to total response rate per question. Chi-Square analyses were used to analyze Benefit and Concern statements using SAS version 9.3.

## Results and discussion

A total of 168 respondents replied to the 2021 survey with a percent completion rate of 84.5%.

**Fig 1:**
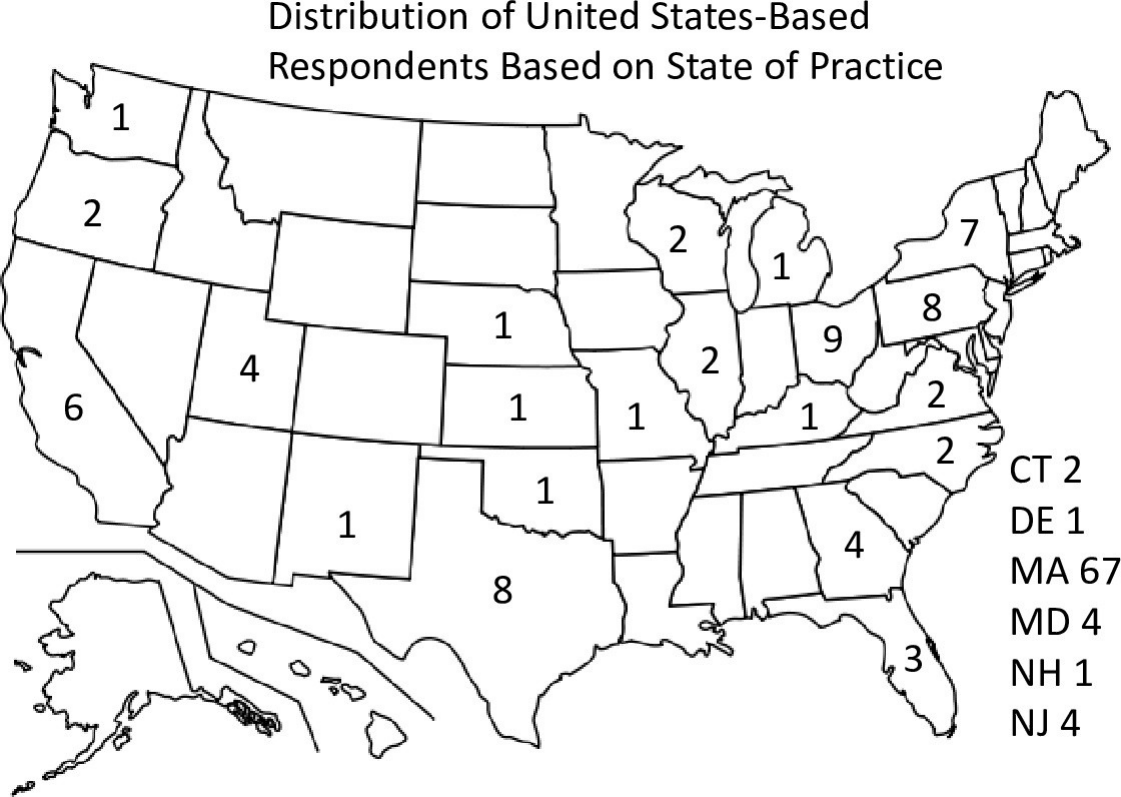
Distribution of United States-Based Respondents Based on State of Practice. Figure developed based on an image that is in the public domain, https://en.wikipedia.org/wiki/File:Blank_US_map_borders.svg [17].

Of the 168 respondents, 94 (60%) identified as male and, 60 (38%) identified as female. Almost three out of four participants (74%) self-identified as white, whereas 6 (4%) as black or African American, 23 (15%) as Asian, 2 (1%) as American Indian or Native Alaskan, and 12 (8%) preferred not to answer. Of the respondents, 75 (49%) have been in practice for over 20 years, while 21 (13%) have been in practice for 5 years or less (Table 1). Massachusetts had the highest number of respondents with 44%, followed by Ohio (5%), Pennsylvania and Texas (4%) (Fig 1). In terms of profession, 91 (54%) were physicians, 15 (10%) were advanced practice providers, 12 (8%) were registered nurses, and 37 (24%) identified as ‘other’. Two thirds of respondents were employed in hospitals or clinics.

**Table 1:** Demographics of survey respondents from the 2021 POCT survey.

Participant Demographics	Number of Respondents (%)
**Gender**	
Male	94 (60)
Female	60 (38)
Undisclosed	3 (2)
**Race**	
White	113 (74)
Black or African American	6 (4)
Asian	23 (15)
American Indian or Native Alaskan	2 (1)
Prefer Not to answer	12 (8)
**Years in Practice**	
0–5 years	21 (14)
6–10 years	14 (9)
11–15 years	24 (16)
16–20 years	20 (13)
Over 20 years	75 (49)
**Profession**	
Physician (MD- Doctor of Medicine/DO- Doctor of Osteopathic Medicine)	91 (59)
Advanced Practice Providers (NP- Nurse Practitioner/ APN- Advanced Practice Nurse/ PA-Physician Assistant)	15 (10)
RN-Registered Nurse	12 (8)
Other	37 (24)
**Patient Practice Environment**	
In-hospital	68 (44)
Ambulatory Clinic	50 (32)
In-home	11 (7)
ER- Emergency Room	6 (4)
Other	21 (14)

### Distribution of specialty

An array of specialties that were reported by the 2021 survey is presented in Table 2. Other specialties included Critical Care, OB-GYN, Psychiatry, and Anesthesiology. The distribution of specialties was similar to those of the 2019–2020 surveys [8,9].

**Table 2:** Specialties represented among survey respondents 2019–2021.

Specialty	Number of Respondents (% of Respondents) 2019	Number of Respondents 2020	Number of Respondents 2021
Family or Internal Medicine	23 (16)	39 (14)	36 (23)
Pulmonology	11 (8)	71 (25)	26 (17)
Emergency Medicine	22 (16)	19 (7)	18 (12)
Cardiology	24 (17)	48 (17)	17 (11)
Hematology	4 (3)	4 (1)	5 (3)
Sleep Medicine	8 (6)	12 (4)	5 (3)
Other	59 (42)	111 (40)	65 (42)

### Important aspects of POCT

Respondents were asked to select the first, second, and third most important characteristic of POCTs incorporated in current practice (Table 3). The numbers and ranks shown in the table were determined by the following point system; 1st most important = 3 points, 2nd most important = 2 points, and 3rd most important = 1 point.

**Table 3:** Survey values^*^ of the important characteristics of point-of-care technology from years 2019–2021.

Characteristic	2019 (n = 154) Weighted points / rank	2020 (n = 287) Weighted points / rank	2021 (n = 168) Weighted points / rank
Accuracy	325 / 1	651 / 1	386 / 1
Ease of Use	175 / 2	345 / 2	195 / 2
Availability	90 / 4	177 / 3	141 / 3
Cost	89 / 5	150 / 4	80 / 4
Reimbursement for Testing	36 / 6	85 / 6	40 / 5
Does not Disturb Workflow	93 / 3	139 / 5	24 / 6
CLIA-waived Status	4 / 10	31 / 10	23 / 7
Sample Collection	18 / 9	32 / 9	21 / 8
Information Systems Connectivity	21 / 8	44 / 7	15 / 9
Sample Type	29 / 7	35 / 8	14 / 10
Ruggedness	4 / 11	6 / 11	9 / 11
Device Footprint	1 / 12	1 / 12	2 / 12

^*^These values were determined with the following point system; 1st most important = 3 points, 2nd most important = 2 points, and 3rd most important = 1 point.

In 2021, the top three characteristics were Accuracy (386), Ease of Use (195), and Availability (141). In 2020 and 2019 the characteristics were similarly chosen, with an exception of Does not Disturb Workflow being in top 3 for 2019. The least frequently chosen characteristics in the years 2019 and 2020 were Device Footprint, Ruggedness, and CLIA (Clinical Laboratory Improvement Amendments)-waived status. In 2021, the lowest chosen characteristics were Device Footprint, Ruggedness, and Sample Type.

### Benefits of POCT

**Fig 2:**
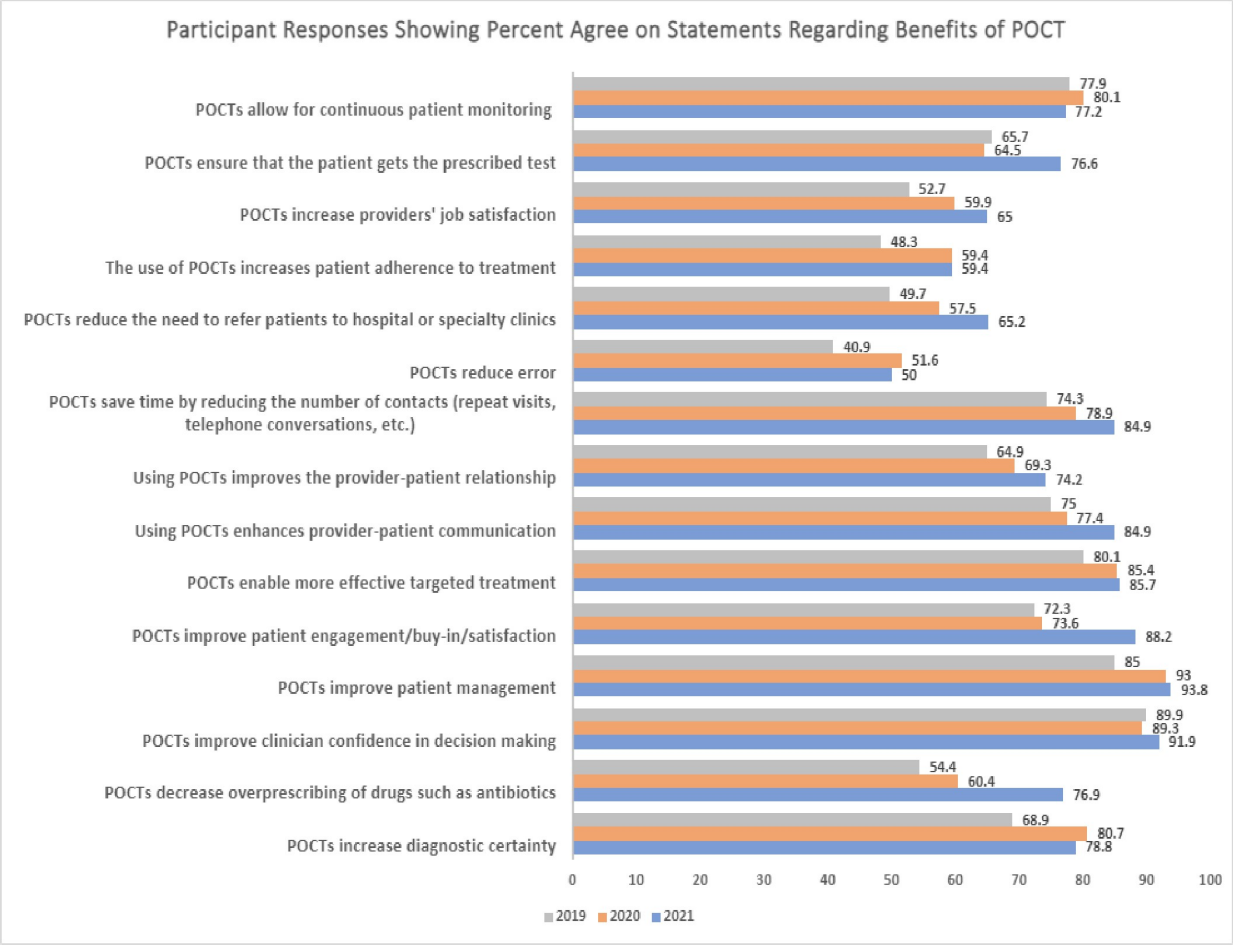
Participant responses showing percent agree on statements regarding benefits of POCT. Comparison of participant responses to the statements regarding the benefits of POCT from years 2019–2021. Respondents were given 15 statements regarding the potential benefits of POCT, and were asked to respond whether they “strongly agreed”,”agreed”, “neutral/ not sure”, “disagreed”, or “strongly disagreed”. The percentages shown reflect those who said they agree or strongly agree with each statement.

Participants were also given a series of 15 statements regarding the benefits of POCT and asked to rate the degree to which they agreed with the statements (Fig 2). In 2021, the top three most agreed upon statements were that POCTs improve patient management (93.8%), improve clinician confidence in decision making (91.9%), and improve patient engagement/buy-in/satisfaction (88.2%). In 2020, the top three statements chosen were POCTs improve patient management (93%), improve clinician confidence in decision making (89.3%), and enable more effective targeted treatment (85.4%). Similarly in 2019 the top three statements were POCTs improve clinician confidence in decision making (89.9%), improve patient management (85%), and enable more effective targeted treatment (80.1%). The least agreed upon statement for all three years with respect to benefits of POCTs was that they reduce error.

Response distribution for most items were similar across the three survey years but there were exceptions. Compared to previous years, the 2021 survey revealed a greater agreement on the benefits of POCTs (Table 4). More respondents in 2021 agreed or strongly agreed that POCTs improved patient management and engagement than previous surveys. The most significant response was a higher agreement that POCTs decreased overprescribing of drugs such as antibiotics (P = <0.0001) in 2021 compared to 2020 and 2019.

**Table 4:** Changes over time between 2019 and 2021 regarding the benefits of POCTs among clinician repondents. Greater agreement was observed in each statement presented in the table.

Benefits	*p*-value^a^
POCTs increase diagnostic certainty	0.0185
POCTs decrease overprescribing of drugs such as antibiotics	< .0001
POCTs improve patient management	0.0093
POCTs improve patient engagement/buy-in/satisfaction	0.0005
POCTs reduce the need to refer patients to hospital or specialty clinics	0.023
POCTs ensure that the patient gets the prescribed test	0.027

^a^Chi-square analysis was used to generate these values.

In the 2021 survey, we tested 5 new statements regarding benefits of POCTs. The statements and the percentage of respondents that agreed (agreed or stronlgy agreed) were as follows: Faster turnaround time with POCT test results increases the opportunity for immediate feedback by a health care provider (93.6%), I am confident a POCT used by a patient at home will produce results as accurate as a POCT used by a provider in a clinical site (39%), an advantage of a POCT is a decreased need for additional patient travel to a blood collection site for central lab testing (84.9%), POCT fingerstick blood test results can be as clinically useful as test results from a venous blood draw sent to a central lab (67.3%), and environmental hygiene and bloodborne pathogen exposure during specimen collection and handling for POCT can be as safe as that of venous blood draw procedures for central laboratory testing (70.1%).

### Concerns regarding POCT among healthcare professionals

**Fig 3:**
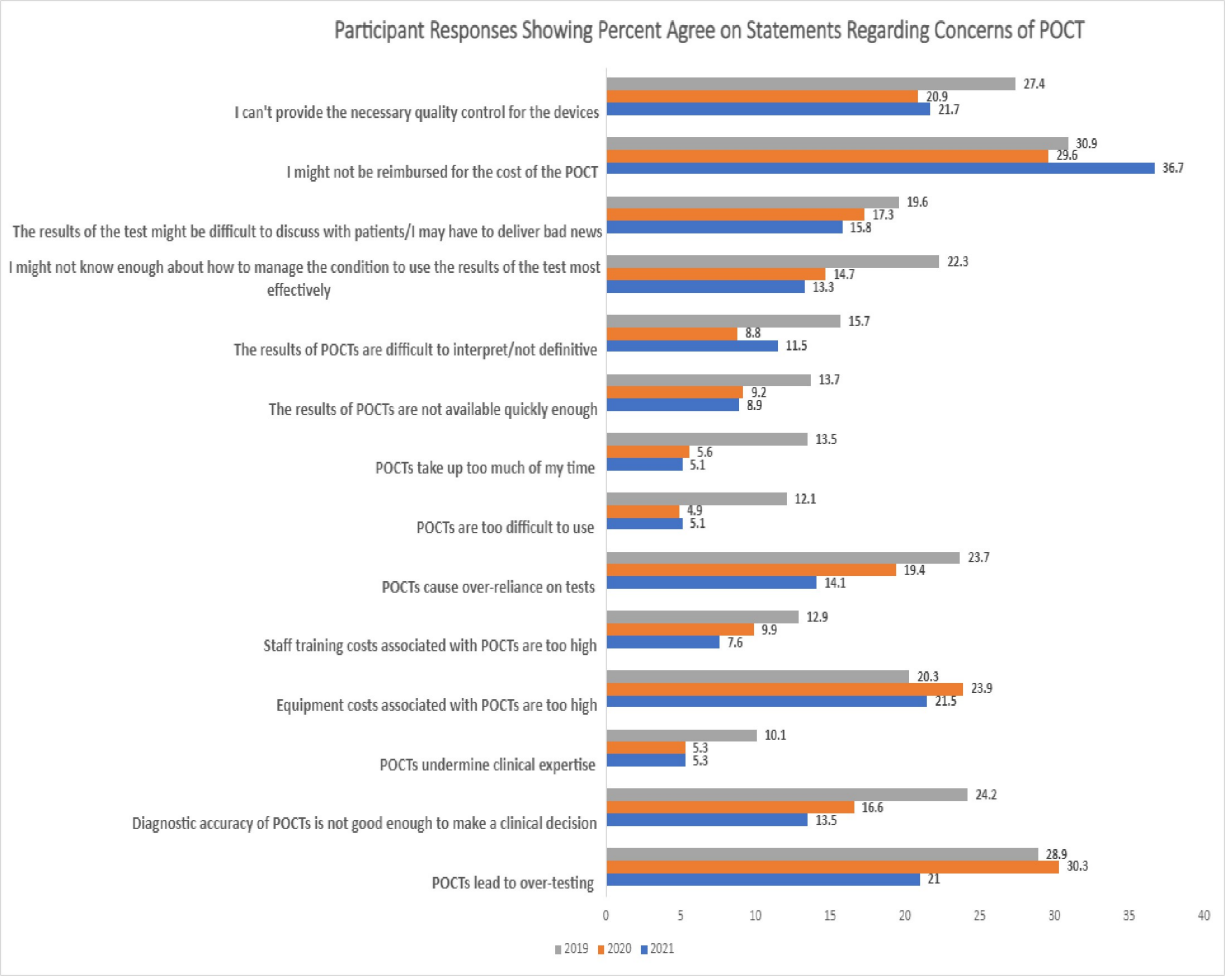
Participant responses showing percent agree statements regarding concerns of POCT. Comparison of participant responses to the statements regarding the concerns of POCT from years 2019–2021. Respondents were given 14 statements regarding the potential benefits of POCT, and were asked to respond whether they “strongly agreed”,”agreed”, “neutral/ not sure”, “disagreed”, or “strongly disagreed”. The percentages shown reflect those who said they agree or strongly agree to the right of each statement.

Participants were given a series of 14 statements regarding the concerns of POCT and asked to rate the degree to which they agreed with the statements of concern. (Fig 3) In 2021, the top three agreed upon concerns were, I might not be reimbursed for the cost of POCT (36.7%), I can’t provide the necessary quality control for the devices (21.7%), and equipment costs associated with POCTs are too high (21.5%). Previous year surveys had similar concerns but in 2020 and 2021, POCTs lead to over-testing was also a concern (30.3% and 28.9%, respectively). Approximately 44% of participants agreed with the statement, “I am concerned about the accuracy of some commercial POCT that my patients use.”

Fewer respondents in 2021 agreed that POCTs were too difficult to use (P = 0.01) or took up too much time (P = 0.005) than in prior surveys (Table 5). In addition there was a decreasing number of respondents from 2019 to 2021 who felt that POCT diagnostic accuracy was not enough to make a clinical decsion (P = 0.04). The most significant concern was POCTs take too much of my time (P = 0.005).

**Table 5:** Changes over time between 2019 and 2021 regarding concerns about POCT among clinician respondents. Less agreement was observed for each statement presented in the table.

Concerns	*p-*value
Diagnostic accuracy of POCTs is not enough to make a clinical decision	0.040
POCTs are too difficult to use	0.011
POCTs take up too much of my time	0.0050

In this manuscript, we report results of surveys distributed to clinicians between 2019 and 2021 to access changes over time in their views about the potential benefits, harms, and key features of POCTs. Our results provide end user perspectives on POCTs to help foster development of the next generation of POCTs that meet the needs of this key group of stakeholders. Most respondents agreed with benefit statements regarding POCTs indicating robust support and an expanding role for these devices. Specifically, POCTs should improve both patient management and clinician confidence in decision making. The percentage agreement during the 3 years remained steady, even throughout the course of the pandemic. When it came to the percentage to which respondents agreed with concerns, results were more variable. In 2019, a higher proportion of healthcare professionals agreed with concern statements, but in 2020 and 2021, a smaller fraction agreed that POCTs were too difficult to use, undermined clinical expertise, and took up too much time. Testing and monitoring of conditions such as diabetes and cardiovascular disease are routinely done at wellness visits, but with limited access to clinics in 2020, less tests were administered [18]. Not only did this limit access to the early diagnosis of these conditions, but it also put patients at risk for developing severe cases of COVID-19 [19]. During the pandemic, states with large numbers of COVID-19–associated deaths also experienced large proportional increases in deaths from other underlying causes [20]. This discovery may have put past perceptions of time required and ease of use of POCTs in perspective for both provider and patient, which may account for the decrease in proportion of respondents that expressed these concerns.

When a provider makes a choice to use or interpret the findings of a POCT device, it is vital to consider the distinct qualities that make it an optimal fit for clinical practice. Furthermore, when faced with multiple POCT options, what makes one POCT preferable over another or a central laboratory test? Accuracy, availability, user-friendliness, or cost-effectiveness can all be influential factors when selecting a test. In this survey, clinician respondents were asked to choose their top 3 most important characteristics when it came to POCTs. Results showed similar characteristics chosen over the years including accuracy, ease of use, availability, and does not disturb workflow. However, in 2020 and 2021, there was an increase in the proportion of respondents who identified the importance of CLIA-waived status (shown in Table 3). CLIA-waived tests are simple tests with a low risk for an incorrect result [21]. In 2019, that characteristic was chosen 7x less by respondents compared to the following years. These results align with findings of prior research conducted by Klepser and colleagues (2021) who reported on increased authorization of US licensed pharmacists to order and administer FDA authorized COVID-19 tests to meet increased patient demand for diagnostic testing [22]. That study determined that pharmacies exhibited a 45% increase of CLIA-waived facilities between 2015 and 2020 (prior and during the pandemic) [22]. This pattern is similarly shown in our data, as a larger proportion of respondents deemed CLIA-waived status being a more important characteristic when it comes to POCT than in prior years.

Although our survey included a diverse group of healthcare professionals from several different specialties, a limitation to our study was its modest sample size that did not include HCPs from every state in the US. This can produce results that are difficult to generalize to broader groups of healthcare providers. Although Internet-survey based research can be efficient in terms of time and cost, it also has its challenges, such as an increase in electronic threats and excessive surveys from various sources, including marketing, customer satisfaction, and political polls [23]. Despite having a monetary incentive, studies have shown this does not always guarantee a successful response rate [23]. In future studies, surveys measuring clinician views of POCT would benefit from a larger and more geographically diverse sample. This could be done by sending to different contacts, specifically survey based research could reach a wider audience by being advertised on multiple social media platforms, which would allow HCPs to share information, educate, and interact with patients, caregivers, students, and colleagues [24]. Future studies can analyze results further by comparing perspectives on POCTs based on profession or specialty to determine if results differ.

## Conclusions

In recent few years there have been notable advancements in POCTs, enabling healthcare practitioners to perform quick diagnostic tests and provide care to patients for a growing number of clinical conditions [10]. The insights derived from our survey results reveals convergence in attitudes towards the benefits of POCT, with respondents expressing a belief in its positive impact on patient management, increased patient engagement, and reduction in overprescribing of antibiotics. Notably, in the 2021 survey, there was statistically significant decrease in concerns related to diagnostic accuracy, ease of use, and time consumption related to POCT. This nuanced understanding of perceptions will inform targeted efforts to enhance and innovate POCT technologies, addressing specific areas of concerns and maximizing their potential benefits. Furthermore, these results demonstrated that an increasing number of respondents believed that POCTs were beneficial, suggesting a growing acknowledgment of their positive impact on healthcare during and after the pandemic. In contrast, concerns of POCT decreased over time, indicating a diminishing level of apprehension about their usage. This shift in attitudes towards POCT reflects an encouraging trend of heightened comfort and confidence among healthcare professionals in incorporating POCTs in their practice. Over the three surveys the most important test characteristics are accuracy, ease of use, and availability. Test developers should keep these characteristics at the top of their mind while commercializing their technologies. With ongoing technological advancements and increasing use of home and over-the-counter POC diagnostics, it is important to maintain a comprehensive understanding of how clinicians view these technologies. To gain further insights, it will be important to conduct surveys in diverse and geographically widespread locations to assess clinician values more robustly regarding the role of POCTs.

## Supporting information

S1 File Clinician facing surveys 2019–2021. (PDF)

S2 File (PDF)

S3 File (PDF)
