# Supplementary material for: Sampling of healthcare professionals’ perspective on point-of-care technologies from 2019–2021: A survey of benefits, concerns, and development
Source: PLoS One. 2024 Mar 8;19(3):e0299516. doi: 10.1371/journal.pone.0299516 (PMC10923439; doi:10.1371/journal.pone.0299516)
Supplement: S2 File — (PDF) [file pone.0299516.s002.pdf]

# Point of Care Testing Survey

Welcome!

Thank you for taking the time to complete our survey. We want your expert opinion about what qualities of point of care technologies (POCTs) are most important to health care providers. Moreover, we would like to better understand the decision-making process of implementing these newer technologies into everyday practice. Our hope is that the results of this survey will identify areas of need and encourage new research and development. Your input will have a direct impact on the technology and use cases we focus on and fund. In this study we are interested in point of care technologies for heart, lung, blood, and sleep diseases.

Point of Care Tests (POCTs) are defined as tests that can be done onsite (in the clinic, ER, home, hospital) with results available during the visit and before the patient leaves. We are asking about POCTs on samples taken from the body, including blood, urine, and other bodily fluids.

This survey was originally released in October 2019 and has been updated in consideration of the COVID-19 pandemic.

This survey should take about 10 minutes. Participation is voluntary and you can stop at any time. Your responses are anonymous. Compensation will be provided for completing the survey ($25).

If you have any questions please email Nate Hafer, PhD at nathaniel.hafer@umassmed.edu

---

This question was placed here only so that REDCap would recognize a section break. It is not relevant to the study and is hidden to prevent responses. ________________________________

**Point of Care Tests (POCTs) are defined as tests that can be done onsite (in the clinic, ER, home, hospital) with results available during the visit and before the patient leaves. We are asking about POCTs on samples taken from the body, including blood, urine, and other bodily fluids. Examples of POCT include blood glucose, blood pressure, EKG, and carbon monoxide breath tests.**

Do you believe that POC testing could improve how you care for patients?

○ strongly agree ○ agree ○ neutral ○ disagree ○ strongly disagree

Name up to 5 conditions for which a POCT could help you make a DIAGNOSIS of a disease. Please list the conditions whether POCTs currently exist for the condition or not.

Condition # 1 ______________________

Condition # 2 ______________________

Condition # 3 ______________________

Condition # 4 ______________________

Condition # 5 ______________________

Name up to 5 conditions for which a POCT could help you MONITOR or MANAGE disease. Please list the conditions whether POCTs currently exist for the condition or not.

Condition # 1 ______________________

Condition # 2 ______________________

Condition # 3 ______________________

Condition # 4 ______________________

Condition # 5 ______________________

Previous studies have identified the following potential BENEFITS of POCTs. To what extent do you think the following aspects of POCTs would be beneficial?

a. POCTs increase diagnostic certainty

◯ strongly disagree ◯ disagree ◯ neutral / not sure ◯ agree ◯ strongly agree

b. POCTs decrease overprescribing of drugs such as antibiotics

◯ strongly disagree ◯ disagree ◯ neutral / not sure ◯ agree ◯ strongly agree

c. POCTs improve clinician confidence in decision making

◯ strongly disagree ◯ disagree ◯ neutral / not sure ◯ agree ◯ strongly agree

d. POCTs improve patient management

◯ strongly disagree ◯ disagree ◯ neutral / not sure ◯ agree ◯ strongly agree

e. POCTs improve patient engagement/buy-in/satisfaction

◯ strongly disagree ◯ disagree ◯ neutral / not sure ◯ agree ◯ strongly agree

f. POCTs enable more effective targeted treatment

◯ strongly disagree ◯ disagree ◯ neutral / not sure ◯ agree ◯ strongly agree

g. Using POCTs enhances provider-patient communication

◯ strongly disagree ◯ disagree ◯ neutral / not sure ◯ agree ◯ strongly agree

h. Using POCTs improves the provider-patient relationship

◯ strongly disagree ◯ disagree ◯ neutral / not sure ◯ agree ◯ strongly agree

i. POCTs save time by reducing the number of contacts (repeat visits, telephone conversations, etc.)

◯ strongly disagree ◯ disagree ◯ neutral / not sure ◯ agree ◯ strongly agree

j. POCTs reduce error

◯ strongly disagree ◯ disagree ◯ neutral / not sure ◯ agree ◯ strongly agree

k. POCTs reduce the need to refer patients to hospital or specialty clinics

◯ strongly disagree ◯ disagree ◯ neutral / not sure ◯ agree ◯ strongly agree

l. The use of POCTs increases patient adherence to treatment

◯ strongly disagree ◯ disagree ◯ neutral / not sure ◯ agree ◯ strongly agree

m. POCTs increase providers' job satisfaction

◯ strongly disagree ◯ disagree ◯ neutral / not sure ◯ agree ◯ strongly agree

n. POCTs ensure that the patient gets the prescribed test

◯ strongly disagree ◯ disagree ◯ neutral / not sure ◯ agree ◯ strongly agree

o. POCTs allow for continuous patient monitoring

◯ strongly disagree ◯ disagree ◯ neutral / not sure ◯ agree ◯ strongly agree

Previous studies have identified the following CONCERNS about POCTs in general. To what extent are you concerned with the following? Please check the most appropriate box for each statement.

a. POCTs lead to over-testing

◯ strongly disagree ◯ disagree ◯ neutral / not sure ◯ agree ◯ strongly agree

b. Diagnostic accuracy of POCTs is not good enough to make a clinical decision

◯ strongly disagree ◯ disagree ◯ neutral / not sure ◯ agree ◯ strongly agree

c. POCTs undermine clinical expertise

◯ strongly disagree ◯ disagree ◯ neutral / not sure ◯ agree ◯ strongly agree

d. Equipment costs associated with POCTs are too high

◯ strongly disagree ◯ disagree ◯ neutral / not sure ◯ agree ◯ strongly agree

e. Staff training costs associated with POCTs are too high

◯ strongly disagree ◯ disagree ◯ neutral / not sure ◯ agree ◯ strongly agree

f. POCTs cause over-reliance on tests

◯ strongly disagree ◯ disagree ◯ neutral / not sure ◯ agree ◯ strongly agree

g. POCTs are too difficult to use

◯ strongly disagree ◯ disagree ◯ neutral / not sure ◯ agree ◯ strongly agree

h. POCTs take up too much of my time

◯ strongly disagree ◯ disagree ◯ neutral / not sure ◯ agree ◯ strongly agree

i. The results of POCTs are not available quickly enough

◯ strongly disagree ◯ disagree ◯ neutral / not sure ◯ agree ◯ strongly agree

j. The results of POCTs are difficult to interpret/not definitive

◯ strongly disagree ◯ disagree ◯ neutral / not sure ◯ agree ◯ strongly agree

k. I might not know enough about how to manage the condition to use the results of the test most effectively

◯ strongly disagree ◯ disagree ◯ neutral / not sure ◯ agree ◯ strongly agree

l. The results of the test might be difficult to discuss with patients/I may have to deliver bad news

◯ strongly disagree ◯ disagree ◯ neutral / not sure ◯ agree ◯ strongly agree

m. I might not be reimbursed for the cost of the POCT

◯ strongly disagree ◯ disagree ◯ neutral / not sure ◯ agree ◯ strongly agree

n. I can't provide the necessary quality control for the devices

◯ strongly disagree ◯ disagree ◯ neutral / not sure ◯ agree ◯ strongly agree

Which characteristic of a point of care technology is most important when incorporating it into your regular practice?

◯ availability
◯ ease of use
◯ accuracy
◯ sample type
◯ sample collection
◯ does not disrupt workflow
◯ cost
◯ device footprint
◯ reimbursement for testing
◯ information systems connectivity
◯ CLIA-waived status
◯ ruggedness

Which characteristic of a point of care technology is the second most important when incorporating it into your regular practice?

◯ availability
◯ ease of use
◯ accuracy
◯ sample type
◯ sample collection
◯ does not disrupt workflow
◯ cost
◯ device footprint
◯ reimbursement for testing
◯ information systems connectivity
◯ CLIA-waived status
◯ ruggedness

Which characteristic of a point of care technology is the third most important when incorporating it into your regular practice?

◯ availability
◯ ease of use
◯ accuracy
◯ sample type
◯ sample collection
◯ does not disrupt workflow
◯ cost
◯ device footprint
◯ reimbursement for testing
◯ information systems connectivity
◯ CLIA-waived status
◯ ruggedness

COVID-19 The next set of questions asks you to provide your thoughts related to POCT and the COVID-19 pandemic.

Rate the extent to which you agree with the following statements.

a. POC testing use is improving patient care during the COVID-19 pandemic.

○ strongly disagree ○ disagree ○ neutral / not sure ○ agree ○ strongly agree

b. POC testing improves diagnosis of patients with COVID-19.

○ strongly disagree ○ disagree ○ neutral / not sure ○ agree ○ strongly agree

c. POC testing has been beneficial in decreasing transmission of COVID-19.

○ strongly disagree ○ disagree ○ neutral / not sure ○ agree ○ strongly agree

d. POCT has increased community access to COVID-19 testing.

○ strongly disagree ○ disagree ○ neutral / not sure ○ agree ○ strongly agree

e. How would you rate your experience with POCT during the COVID-19 pandemic?

○ very poor ○ poor ○ neutral / not sure / no experience ○ good ○ excellent/very good

f. How would you categorize the extent of your experience with POCT during the COVID-19 pandemic?

○ substantial / a lot ○ moderate ○ minimal ○ none

The next set of questions asks that you reflect on the strategic decision-making styles of your practice and/or the hospital or health care setting where you spend the most time.

Rate the extent to which you agree with the following statements characterizing the external business environment within which your practice operates.

My practice operates in a very safe external business environment, with little threat to its survival.

○ strongly disagree ○ disagree ○ neutral / not sure ○ agree ○ strongly agree

My practice's external business environment has few investment opportunities.

○ strongly disagree ○ disagree ○ neutral / not sure ○ agree ○ strongly agree

My practice operates in an external business environment it largely controls (i.e., like a dominant firm in an industry with little competition and few hindrances).

○ strongly disagree ○ disagree ○ neutral / not sure ○ agree ○ strongly agree

Rate the extent to which you agree with the following characterizations of the top management philosophy within your practice.

Practice communication channels are highly structured.

○ strongly disagree ○ disagree ○ neutral / not sure ○ agree ○ strongly agree

There is a strong emphasis on utilizing tried-and-true methods despite changes to business conditions.

◯ strongly disagree ◯ disagree ◯ neutral / not sure ◯ agree ◯ strongly agree

There is loose, informal control for getting work done.

◯ strongly disagree ◯ disagree ◯ neutral / not sure ◯ agree ◯ strongly agree

Rate the extent to which you agree with the following characterizations of the organizational culture within which your practice operates.

It is generally known throughout our practice that our intention is to grow as big and as fast as possible.

◯ strongly disagree ◯ disagree ◯ neutral / not sure ◯ agree ◯ strongly agree

In our search for new opportunities, my practice is primarily driven by changes in society-at-large.

◯ strongly disagree ◯ disagree ◯ neutral / not sure ◯ agree ◯ strongly agree

When considering resources needed to pursue new opportunities, my practice favors heavy and rapid investment in existing resources rather than outsourcing, borrowing, and renting.

◯ strongly disagree ◯ disagree ◯ neutral / not sure ◯ agree ◯ strongly agree

Please characterize your practice's adoption of new lines of heart, lung, blood, and sleep disease products or services over the past 5 years by indicating your agreement with the following statements:

We change product or service lines infrequently.

◯ strongly disagree ◯ disagree ◯ neutral / not sure ◯ agree ◯ strongly agree

Point of care technology is a top priority; risk is accepted to achieve growth.

◯ strongly disagree ◯ disagree ◯ neutral / not sure ◯ agree ◯ strongly agree

Adoption of new lines of products or services is often constrained by available resources.

◯ strongly disagree ◯ disagree ◯ neutral / not sure ◯ agree ◯ strongly agree

Please characterize your practice's relationship with its competitors by rating your agreement with the following statements:

My practice often moves to introduce new products or services, administrative techniques, or operating technologies before seeing what our competition will do.

◯ strongly disagree ◯ disagree ◯ neutral / not sure ◯ agree ◯ strongly agree

My practice actively focuses on taking business away from our competitors.

◯ strongly disagree ◯ disagree ◯ neutral / not sure ◯ agree ◯ strongly agree

My practice engages in co-development, partnerships, or joint ventures with our competitors.

◯ strongly disagree ◯ disagree ◯ neutral / not sure ◯ agree ◯ strongly agree

Please characterize your practice's decision-making tendencies by rating your agreement with the following statements:

My practice's top managers believe that our business environment requires bold, wide-ranging decisions to achieve our objectives.

◯ strongly disagree ◯ disagree ◯ neutral / not sure ◯ agree ◯ strongly agree

My practice typically adopts a cautious, 'wait-and-see' posture in order to minimize the possibility of making costly decisions.

◯ strongly disagree ◯ disagree ◯ neutral / not sure ◯ agree ◯ strongly agree

My practice's top managers evaluate and promote employees based on the value that they add to the practice.

◯ strongly disagree ◯ disagree ◯ neutral / not sure ◯ agree ◯ strongly agree

Demographic Questions

What is your gender?

◯ male
◯ female
◯ other
◯ would rather not say

What is your specialty? (select all that apply)

☐ Cardiology
☐ Family or Internal Medicine
☐ Pulmonology
☐ Hematology
☐ Emergency Medicine
☐ Sleep Medicine
☐ Other

Please describe other specialty:

__________________________________

What is your profession?

◯ MD-Medical Doctor
◯ DO-Doctor of Osteopathy
◯ NP-Nurse Practitioner
◯ APN-Advanced Practice Nurse
◯ PA-Physicians' Assistant
◯ RN-Registered Nurse
◯ Other

Please describe your profession:

__________________________________

Where is your practice located?

◯ Alabama
◯ Alaska
◯ Arizona
◯ Arkansas
◯ California
◯ Colorado
◯ Connecticut
◯ Delaware
◯ District of Columbia
◯ Florida
◯ Georgia
◯ Hawaii
◯ Idaho
◯ Illinois
◯ Indiana
◯ Iowa
◯ Kansas
◯ Kentucky
◯ Louisiana
◯ Maine
◯ Maryland
◯ Massachusetts
◯ Michigan
◯ Minnesota
◯ Mississippi
◯ Missouri
◯ Montana
◯ Nebraska
◯ Nevada
◯ New Hampshire
◯ New Jersey
◯ New Mexico
◯ New York
◯ North Carolina
◯ North Dakota
◯ Ohio
◯ Oklahoma
◯ Oregon
◯ Pennsylvania
◯ Rhode Island
◯ South Carolina
◯ South Dakota
◯ Tennessee
◯ Texas
◯ Utah
◯ Vermont
◯ Virginia
◯ Washington
◯ West Virginia
◯ Wisconsin
◯ Wyoming
◯ Other

Please describe other location: ______________________________

What is your practice environment?

◯ In-home,
◯ ambulatory clinic
◯ ER
◯ in-hospital
◯ other

| | |
|---|---|
| Please specify other practice environment. | __________________________________ |
| What is the best approximation of your primary ambulatory practice? | ◯ Public Health Clinic<br>◯ Federally Qualified Health Center<br>◯ College/University<br>◯ Health Maintenance Organization<br>◯ Private Community Clinic<br>◯ Private-Single Practitioner Setting<br>◯ Private-Multiple Practitioner Setting<br>◯ Hospital-owned Single Group Practice |
| How many years have you practiced after completing your terminal training/degree? | ◯ 0-5 years<br>◯ 6-10 years<br>◯ 11-15 years<br>◯ 16-20 years<br>◯ Over 20 years |
| What is your race? | ☐ White<br>☐ Black or African American<br>☐ Asian<br>☐ American Indian or Alaska Native<br>☐ Native Hawaiian or Other Pacific Islander<br>☐ Other<br>☐ I prefer not to respond |
| Specify other race | __________________________________ |
| What is your ethnicity? | ◯ Hispanic or Latino<br>◯ Not Hispanic or Latino<br>◯ I prefer not to respond |
