# Supplementary material for: Sampling of healthcare professionals’ perspective on point-of-care technologies from 2019–2021: A survey of benefits, concerns, and development
Source: PLoS One. 2024 Mar 8;19(3):e0299516. doi: 10.1371/journal.pone.0299516 (PMC10923439; doi:10.1371/journal.pone.0299516)
Supplement: S3 File — (PDF) [file pone.0299516.s003.pdf]

Point of Care Testing Survey 2021

Welcome!

Thank you for taking the time to complete our survey. We want your expert opinion about what qualities of point of care technologies (POCTs) are most important to health care providers. Moreover, we would like to better understand the decision-making process of implementing these newer technologies into everyday practice. Our hope is that the results of this survey will identify areas of need and encourage new research and development. Your input will have a direct impact on the technology and use cases we focus on and fund. In this study we are interested in point of care technologies for heart, lung, blood, and sleep diseases.

Point of Care Tests (POCTs) are defined as tests that can be done onsite (in the clinic, ER, home, hospital) with results available during the visit and before the patient leaves. We are asking about POCTs on samples or measurements taken from the body, including blood, urine, and other bodily fluids.

This survey should take about 10 minutes. Participation is voluntary and you can stop at any time. Your responses are anonymous. Compensation will be provided for completing the survey ($25).

If you have any questions please email Nate Hafer, PhD at nathaniel.hafer@umassmed.edu

---

This question was placed here only so that REDCap would recognize a section break. It is not relevant to the study and is hidden to prevent responses. ________________________________

Point of Care Tests (POCTs) are defined as tests that can be done onsite (in the clinic, ER, home, hospital) with results available during the visit and before the patient leaves. We are asking about POCTs on samples or measurements taken from the body, including blood, urine, and other bodily fluids. Examples of POCT include blood glucose, blood pressure, EKG, and carbon monoxide breath tests.

Do you believe that POC testing could improve how you care for patients?

◯ strongly agree
◯ agree
◯ neutral
◯ disagree
◯ strongly disagree

Do you believe that the use of POC testing has changed since the start of the COVID-19 pandemic?

◯ greatly increased
◯ somewhat increased
◯ no change
◯ somewhat decreased
◯ greatly decreased

Do you believe that POC testing has affected health equity?

◯ Yes
◯ No

Has POC testing reduced or increased health inequities?

◯ greatly reduced
◯ somewhat reduced
◯ no difference
◯ somewhat increased
◯ greatly increased

Name up to 5 conditions for which a POCT could help you make a DIAGNOSIS of a disease. Please list the conditions whether POCTs currently exist for the condition or not.

Condition # 1 ________________________________

Condition # 2 ________________________________

Condition # 3 ________________________________

Condition # 4 ________________________________

Condition # 5 ________________________________

Name up to 5 conditions for which a POCT could help you MONITOR or MANAGE disease. Please list the conditions whether POCTs currently exist for the condition or not.

Condition # 1 ________________________________

Condition # 2 ________________________________

Condition # 3 ________________________________

Condition # 4 __________________________________

Condition # 5 __________________________________

Previous studies have identified the following potential BENEFITS of POCTs. To what extent do you think the following aspects of POCTs would be beneficial?

	strongly disagree	disagree	neutral / not sure	agree	strongly agree
a. POCTs increase diagnostic certainty	○	○	○	○	○
b. POCTs decrease overprescribing of drugs such as antibiotics	○	○	○	○	○
c. POCTs improve clinician confidence in decision making	○	○	○	○	○
d. POCTs improve patient management	○	○	○	○	○
e. POCTs improve patient engagement/buy-in/satisfaction	○	○	○	○	○
f. POCTs enable more effective targeted treatment	○	○	○	○	○
g. Using POCTs enhances provider-patient communication	○	○	○	○	○
h. Using POCTs improves the provider-patient relationship	○	○	○	○	○
i. POCTs save time by reducing the number of contacts (repeat visits, telephone conversations, etc.)	○	○	○	○	○
j. POCTs reduce error	○	○	○	○	○
k. POCTs reduce the need to refer patients to hospital or specialty clinics	○	○	○	○	○
l. The use of POCTs increases patient adherence to treatment	○	○	○	○	○
m. POCTs increase providers' job satisfaction	○	○	○	○	○
n. POCTs ensure that the patient gets the prescribed test	○	○	○	○	○
o. POCTs allow for continuous patient monitoring	○	○	○	○	○

p. Faster turnaround time with POC test results increases the opportunity for immediate feedback by a health care provider	○	○	○	○	○
q. I am confident a POCT used by a patient at home will produce results as accurate as a POCT used by a provider in a clinical site	○	○	○	○	○
r. An advantage of a POCT is a decreased need for additional patient travel to a blood collection site for central lab testing	○	○	○	○	○
s. POCT fingerstick blood test results can be as clinically useful as test results from a venous blood draw sent to a central lab	○	○	○	○	○
t. Environmental hygiene and bloodborne pathogen exposure during specimen collection and handling for POCT can be as safe as that of venous blood draw procedures for central laboratory testing	○	○	○	○	○

Previous studies have identified the following CONCERNS about POCTs in general. To what extent are you concerned with the following? Please check the most appropriate box for each statement.

	strongly disagree	disagree	neutral / not sure	agree	strongly agree
a. POCTs lead to over-testing	○	○	○	○	○
b. Diagnostic accuracy of POCTs is not good enough to make a clinical decision	○	○	○	○	○
c. POCTs undermine clinical expertise	○	○	○	○	○
d. Equipment costs associated with POCTs are too high	○	○	○	○	○
e. Staff training costs associated with POCTs are too high	○	○	○	○	○

f. POCTs cause over-reliance on tests	○	○	○	○	○
g. POCTs are too difficult to use	○	○	○	○	○
h. POCTs take up too much of my time	○	○	○	○	○
i. The results of POCTs are not available quickly enough	○	○	○	○	○
j. The results of POCTs are difficult to interpret/not definitive	○	○	○	○	○
k. I might not know enough about how to manage the condition to use the results of the test most effectively	○	○	○	○	○
l. The results of the test might be difficult to discuss with patients/I may have to deliver bad news	○	○	○	○	○
m. I might not be reimbursed for the cost of the POCT	○	○	○	○	○
n. I can't provide the necessary quality control for the devices	○	○	○	○	○
o. I am concerned about the accuracy of some commercial POCT that my patients use	○	○	○	○	○

What characteristic of a point of care technology is 1st, 2nd, and 3rd most important when incorporating it into your regular practice?

	1st most important	2nd most important	3rd most important
1. availability	○	○	○
2. ease of use	○	○	○
3. accuracy	○	○	○
4. sample type	○	○	○
5. sample collection	○	○	○
6. does not disrupt workflow	○	○	○
7. cost	○	○	○
8. device footprint	○	○	○
9. reimbursement for testing	○	○	○
10. information systems connectivity	○	○	○
11. CLIA-waived status	○	○	○
12. ruggedness	○	○	○

The next set of questions asks that you reflect on the strategic decision-making styles of your practice and/or the hospital or health care setting where you spend the most time.

Rate the extent to which you agree with the following statements characterizing the external business environment within which your practice operates.

	strongly disagree	disagree	neutral / not sure	agree	strongly agree
My practice's external business environment has few investment opportunities.	○	○	○	○	○

Rate the extent to which you agree with the following characterizations of the top management philosophy within your practice.

	strongly disagree	disagree	neutral / not sure	agree	strongly agree
Practice communication channels are highly structured.	○	○	○	○	○
There is a strong emphasis on utilizing tried-and-true methods despite changes to business conditions.	○	○	○	○	○

Rate the extent to which you agree with the following characterizations of the organizational culture within which your practice operates.

	strongly disagree	disagree	neutral / not sure	agree	strongly agree
It is generally known throughout our practice that our intention is to grow as big and as fast as possible.	○	○	○	○	○
In our search for new opportunities, my practice is primarily driven by changes in society-at-large.	○	○	○	○	○
When considering resources needed to pursue new opportunities, my practice favors purchasing rather than outsourcing, borrowing, or renting.	○	○	○	○	○

Please characterize your practice's adoption of new lines of heart, lung, blood, and sleep disease products or services over the past 5 years by indicating your agreement with the following statements:

	strongly disagree	disagree	neutral / not sure	agree	strongly agree
We change product or service lines infrequently.	○	○	○	○	○
Point of care technology is a top priority; risk is accepted to achieve growth.	○	○	○	○	○

Please characterize your practice's relationship with its competitors by rating your agreement with the following statements:

	strongly disagree	disagree	neutral / not sure	agree	strongly agree
My practice often moves to introduce new products or services, administrative techniques, or operating technologies before seeing what our competition will do.	◯	◯	◯	◯	◯
My practice engages in co-development, partnerships, or joint ventures with our competitors.	◯	◯	◯	◯	◯

Please characterize your practice's decision-making tendencies by rating your agreement with the following statements:

	strongly disagree	disagree	neutral / not sure	agree	strongly agree
My practice's top managers believe that our business environment requires bold, wide-ranging decisions to achieve our objectives.	◯	◯	◯	◯	◯
My practice typically adopts a cautious, 'wait-and-see' posture in order to minimize the possibility of making costly decisions.	◯	◯	◯	◯	◯

Demographic Questions

What is your gender?

- ◯ male
- ◯ female
- ◯ other
- ◯ would rather not say

What is your specialty? (select all that apply)

- ☐ Cardiology
- ☐ Family or Internal Medicine
- ☐ Pulmonology
- ☐ Hematology
- ☐ Emergency Medicine
- ☐ Sleep Medicine
- ☐ Other

Please describe other specialty:

____

What is your profession?

- ◯ MD-Medical Doctor
- ◯ DO-Doctor of Osteopathy
- ◯ NP-Nurse Practitioner
- ◯ APN-Advanced Practice Nurse
- ◯ PA-Physicians' Assistant
- ◯ RN-Registered Nurse
- ◯ Other

Please describe your profession:

____

What state is your practice located in?

- ◯ Alabama
- ◯ Alaska
- ◯ Arizona
- ◯ Arkansas
- ◯ California
- ◯ Colorado
- ◯ Connecticut
- ◯ Delaware
- ◯ District of Columbia
- ◯ Florida
- ◯ Georgia
- ◯ Hawaii
- ◯ Idaho
- ◯ Illinois
- ◯ Indiana
- ◯ Iowa
- ◯ Kansas
- ◯ Kentucky
- ◯ Louisiana
- ◯ Maine
- ◯ Maryland
- ◯ Massachusetts
- ◯ Michigan
- ◯ Minnesota
- ◯ Mississippi
- ◯ Missouri
- ◯ Montana
- ◯ Nebraska
- ◯ Nevada
- ◯ New Hampshire
- ◯ New Jersey
- ◯ New Mexico
- ◯ New York
- ◯ North Carolina
- ◯ North Dakota
- ◯ Ohio
- ◯ Oklahoma
- ◯ Oregon
- ◯ Pennsylvania
- ◯ Rhode Island
- ◯ South Carolina
- ◯ South Dakota
- ◯ Tennessee
- ◯ Texas
- ◯ Utah
- ◯ Vermont
- ◯ Virginia
- ◯ Washington
- ◯ West Virginia
- ◯ Wisconsin
- ◯ Wyoming
- ◯ Other

Please describe other location:

____

What is your practice environment?	◯ In-home<br>◯ ambulatory clinic<br>◯ ER<br>◯ in-hospital<br>◯ other
Please specify other practice environment.	__________________________
What is the best approximation of your primary ambulatory practice?	◯ Public Health Clinic<br>◯ Federally Qualified Health Center<br>◯ College/University<br>◯ Health Maintenance Organization<br>◯ Private Community Clinic<br>◯ Private-Single Practitioner Setting<br>◯ Private-Multiple Practitioner Setting<br>◯ Hospital-owned Single Group Practice
How many years have you practiced after completing your terminal training/degree?	◯ 0-5 years<br>◯ 6-10 years<br>◯ 11-15 years<br>◯ 16-20 years<br>◯ Over 20 years
What is your race?	☐ White<br>☐ Black or African American<br>☐ Asian<br>☐ American Indian or Alaska Native<br>☐ Native Hawaiian or Other Pacific Islander<br>☐ Other<br>☐ I prefer not to respond
Specify other race	__________________________
What is your ethnicity?	◯ Hispanic or Latino<br>◯ Not Hispanic or Latino<br>◯ I prefer not to respond
